# Zbtb7b defines a compensatory mechanism in MASLD‐related HCC progression by suppressing H19‐mediated hepatic lipid deposition

**DOI:** 10.14814/phy2.70160

**Published:** 2024-12-23

**Authors:** Yinglin Han, Kaimin Wu, Xin Peng, Yinkun Fu, Wenyan Li, Jing Ma, He Jiang, Xu‐Yun Zhao

**Affiliations:** ^1^ Department of Biochemistry and Molecular Cell Biology, Shanghai Key Laboratory for Tumor Microenvironment and Inflammation, Key Laboratory of Cell Differentiation and Apoptosis of National Ministry of Education Shanghai Jiao Tong University School of Medicine Shanghai China; ^2^ Department of Pathophysiology, Key Laboratory of Cell Differentiation and Apoptosis of Ministry of Education Shanghai Jiao Tong University School of Medicine Shanghai China; ^3^ Department of Endocrinology and Metabolism, Renji Hospital Shanghai Jiao Tong University School of Medicine Shanghai China; ^4^ Liver Cancer Institute, Zhongshan Hospital Fudan University, Shanghai Medical College Shanghai China

**Keywords:** de novo lipogenesis, fatty acid oxidation, H19, hepatocellular carcinoma, metabolic dysfunction‐associated steatotic liver disease, Zbtb7b

## Abstract

Hepatocellular carcinoma (HCC) is a widely prevalent type of primary liver cancer. However, strategies for pretumor intervention are still limited. In this study, a liver‐specific Zbtb7b knockout mouse model was used to evaluate the role of Zbtb7b in metabolic dysfunction‐associated steatotic liver disease (MASLD)‐related HCC development. We revealed that Zbtb7b was compensatively increased and restricted lipid deposition in the liver during MASLD progression, which protects against MASLD‐related HCC initiation. Mechanistically, Zbtb7b suppresses the expression of the long noncoding RNA H19 to attenuate hepatic de novo lipogenesis and increase fatty acid oxidation, thereby preventing lipid accumulation in hepatocytes. As a result, the proliferation and migration abilities of HCC cells are reduced. Overall, we demonstrated that Zbtb7b serves as a tumor suppressor at an early stage of HCC, thus providing a promising target for the treatment of HCC at a premalignant stage.

## Graphical abstract

Zbtb7b is compensatively increased during progression of MASLD and restricts lipid deposition in the liver by regulating H19‐mediated de novo lipogenesis and fatty acid oxidation, which protects against MASLD‐related HCC initiation.

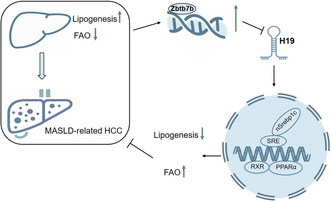

## 1 INTRODUCTION

Liver cancer, including hepatocellular carcinoma (HCC), is a major health challenge with high incidence and mortality rates worldwide (Sia et al., 2017). HCC accounts for 90% of liver cancer cases and has become the fourth leading cause of cancer‐related death globally (Anwanwan et al., 2020; Ringelhan et al., 2018). However, given the insidious onset, high invasiveness and metastasis of liver cancer, most patients are diagnosed at an advanced stage (Gingold et al., 2018), making a huge difficulty in systemic therapies. Additionally, current targeted strategies, such as immunotherapies, have limitations in terms of drug resistance, therefore, significant improvements in the survival rates and prognosis of HCC patients have hardly been achieved (Llovet et al., 2021; Villanueva, 2019). As such, novel preventive measures and effective treatments at an early stage of HCC are urgently needed.

The liver is a vital organ that manages whole‐body metabolic homeostasis. Dysregulation of hepatic lipid metabolism leads to the onset of metabolic dysfunction‐associated steatotic liver disease (MASLD, formerly NAFLD), which is associated with a high risk of progression to HCC (Martínez‐Reyes & Chandel, 2021; Satriano et al., 2019). In recent years, the importance of lipid metabolic reprogramming in the carcinogenic process has become increasingly clear (Currie et al., 2013; Du et al., 2022). Fatty acids not only serve as energy sources to increase the proliferation of cancer cells but also act as signaling molecules to facilitate mitogenic and/or oncogenic signaling (Nakagawa et al., 2018; Zhu & Thompson, 2019). The abundance and desaturation of fatty acids as well as the elongation of fatty acid chains are closely related to the occurrence and development of various tumors (Hoy et al., 2021). Interestingly, normal cells primarily acquire lipids through external uptake, whereas enhanced de novo fatty acid synthesis and suppressed fatty acid oxidation widely occur in tumor cells and largely contribute to tumorigenesis, especially in HCC (Ning et al., 2022, Wang, Han, et al., 2016, Seo et al., 2020, Bian et al., 2024). Sterol regulatory element‐binding protein 1c (Srebp1c) is a master regulator of de novo lipogenesis in the liver, which transcriptionally induces the expression of the lipogenic enzymes fatty acid synthase (Fasn) and stearoyl‐CoA desaturase 1 (Scd1) to drive the de novo lipogenic program (Pope & Dixon, 2023; Yi et al., 2020). On the other hand, peroxisome proliferator‐activated receptor α (Pparα) enhances the transcription of genes encoding rate‐limiting enzymes required for fatty acid oxidation, such as enoyl‐CoA hydratase and 3‐hydroxyacyl CoA dehydrogenase (Ehhadh) and 3‐hydroxy‐3‐methylglutaryl‐CoA synthase 2 (Hmgcs2), in hepatocytes (Li et al., 2024).

Zinc finger and BTB domain containing 7B (Zbtb7b), a member of the POK family of transcriptional repressors, possesses a 7B zinc finger domain that facilitates DNA binding and a POZ/BTB domain to modulate interactions with other factors (Taniuchi, 2018). The major physiological function of Zbtb7b has been demonstrated as a master regulator of lineage commitment in CD4^+^ T cells. However, several studies have shown that Zbtb7b unexpectedly plays an indispensable role in the formation of multiple types of tumors, nevertheless, the underlying mechanisms are not completely understood (Chen et al., 2022; He et al., 2005; Lee et al., 2015; Mariani et al., 2013). Interestingly, a recent study revealed that Zbtb7b deficiency downregulates the fatty acid oxidation pathway in brown fat, resulting in increased fat accumulation and cold sensitivity (Li et al., 2017). However, the role of Zbtb7b in MASLD progression and MASLD‐related HCC development remains elusive.

Here, we revealed that Zbtb7b is a suppressor of MASLD‐related HCC. Zbtb7b deficiency in mouse hepatocytes increases de novo lipogenesis while hepatic fatty acid oxidation is inhibited. Consequently, lipid deposition in the liver is increased, which facilitates the progression of MASLD, and eventually develops to HCC. Mechanistically, depletion of Zbtb7b drastically induced long noncoding RNA H19 expression, thereby driving hepatic de novo lipogenesis and suppressing the fatty acid oxidation program to accelerate MASLD‐related HCC progression.

## 2 MATERIALS AND METHODS

### 2.1 Bioinformatics analysis

GEPIA (https://gepia.cance r‐pku.cn/) was used to reveal the differences in the expression of Zbtb7b and H19 between HCC tissues (*n* = 369) and normal tissues (*n* = 50) in The Cancer Genome Atlas (TCGA) project. Correlations between Zbtb7b or H19 and genes involved in lipid metabolism in HCC tissues in the TCGA project were also analyzed via GEPIA.

### 2.2 Human samples and study approval

All human HCC and adjacent peritumoral tissue samples in our study were obtained from Zhongshan Hospital, and informed consent was obtained from the participants. The research was prospectively reviewed and approved by the Ethics Committee of Zhongshan Hospital (Reference Number: 2022BAT6946). All the research was conducted in accordance with both the Declarations of Helsinki and Istanbul.

### 2.3 AAV production

The production of adeno‐associated viruses (AAVs) involves four procedures: plasmid preparation, virus packaging, virus purification, and titer determination. First, three types of plasmids were prepared to package viruses, including the target plasmid and helper plasmid pAdDeltaF6 (DF6), which can provide E2a, E4, VARNA, and serotype plasmids selected based on organ affinity. In our study, the liver‐specific serotype AAV2/8 was used (Liu et al., 2023; Zhao et al., 2018). When the cell confluence reached 95%, 70 μg of the target plasmid, 200 μg of DF6 and 70 μg of AAV2/8, along with 1360 μL of polyethyleneimine were transferred to ten 15 cm dishes, and the precipitates were harvested by cell scrapers and centrifugation 60 h later. Finally, high‐titer viral solutions were obtained through virus concentration, density gradient centrifugation and column purification (Amicon Ultra 15, Millipore, UFC9100960) steps. In details, we concentrated the virus through three freeze/thaw cycles and centrifugation, performed density gradient centrifugation with different concentrations of iodixanol solution (BasalMedia, R714JV) and purified using purification columns with a phosphate‐buffered saline (PBS) solution containing 10^−3^ F188 (Sigma, P5556‐100ML) and a PBS solution. The obtained viral titer was approximately between 1 × 10^12^ and 1 × 10^13^ vp/mL.

### 2.4 Animals

The mice were raised in a specific pathogen‐free environment, with all the mice having a C57BL/6J strain background. The experiments were performed with rigorous control of age and gender and strict adherence to procedures approved by the University Committee on the Use and Care of Animals at Shanghai Jiao Tong University (Reference Number: A2022‐064). All the mice were euthanized via carbon dioxide (CO_2_) inhalation after the experiments were completed. Zbtb7b^flox/flox^ mice were obtained from R. Bosselut from the National Cancer Institute and fed regular rodent chow. The mice were randomly assigned to groups for injection of the AAV virus and subsequently raised under the same conditions. For the high‐fat diet (HFD) feeding experiment, male Zbtb7b^flox/flox^ mice at 8 weeks of age were injected via the tail vein with 1×10^11^ AAV2/8‐TBG‐Cre viral particles to delete Zbtb7b specifically in the liver (ZLKO). After 7 days, the mice were fed a rodent diet with 60 kcal% fat (D12492, Research diet), and the weights of the mice were measured weekly. After 5 months, the mice were euthanized, and the livers were harvested and photographed. For the MASLD‐related HCC experiment, male ZLKO mice were fed an L‐amino acid diet with 45 kcal% fat with 0.1% methionine and no added choline (CDA‐HFD, A06071309, Research diet) for 7 months, and the livers of the mice were harvested and photographed. For the Akt/N‐Ras‐induced HCC experiment, 7 days after AAV injection, liver cancer oncogenes (pT3‐Nras/pT3‐Akt) along with Sleeping Beauty transposase (SB100) were injected via hydrodynamic transfection (HDT) into WT and ZLKO male mice (Sun et al., 2017). Liver tissues were harvested after 4 weeks to determine the tumor burden.

### 2.5 Plasmid production

The target gene fragments were obtained via PCR from cDNA retro‐transcribed from human HCC tissue RNA and then inserted into the pMSCV destination vector using 2 × ClonExpress Mix (Vazyme, C115‐01) according to the manufacturer's instructions. The integrity and fidelity of all the constructs were verified via DNA sequencing.

### 2.6 Liver histology

Tissues were dissected and fixed in 10% formalin overnight at 4°C and subjected to paraffin embedding and H&E staining. Liver slices were fixed in O.C.T. (Servicebio, G6059‐110ML) and then stained with Oil Red O (ORO) (Sigma, O0625). For Sirius Red staining, the paraffin sections were dewaxed and hydrated, stained with picrosirius red for 1 h, washed with two changes of acidified water, dehydrated and cleared in xylene before mounting. For immunohistochemistry (IHC) assay, the paraffin sections were dewaxed and hydrated. Antigen retrieval was then performed by incubating the sections with EDTA (AiFang biological, AFIHC008). Next, the slices were incubated in a 3% hydrogen peroxide solution, blocked with 3% BSA (Servicebio, G5001) and incubated with primary antibodies F4/80 (Proteintech, 28,463‐1‐AP) overnight at 4°C. On the second day, the slices were incubated with horseradish peroxidase‐conjugated goat anti‐rabbit secondary antibody (AiFang biological, AFIHC003) at room temperature for 30 min. Then DAB chromogen solution (AiFang biological, AFIHC004) was added and the slices were subjected to a 3‐min with hematoxylin. Subsequent immersion in the bluing solution led to the restoration of a blue coloration in the hematoxylin. Finally, the slices were dehydrated and sealed.

### 2.7 Cell lines

AML12, Huh7 and HepG2 cells were obtained from the ATCC. AML12 cells were cultured in DMEM/F‐12 (BasalMedia, L310KJ) supplemented with 10% fetal bovine serum (Sigma, F2442), 1% penicillin streptomycin (BasalMedia, S110), 0.45% ITS Liquid Media Supplement (Sigma, I3146) and 40 ng/mL dexamethasone (Sigma, D2915). Huh7 and HepG2 cells were cultured in DMEM (BasalMedia, L110KJ) supplemented with 10% fetal bovine serum and 1% penicillin streptomycin. All the cells were maintained in a humidified chamber at 37°C under a 5% CO_2_ atmosphere. For the generation of ZBTB7B‐ and H19‐overexpressing cell lines, retroviral transduction was performed to express target genes. For the generation of *ZBTB7B* knockout Huh7 cell lines, sgRNA sequences with minimal off‐target effects on *ZBTB7B* exons were designed via CRISPOR (tefor.net), subsequently, plasmids containing selected sgRNA sequences were constructed, and their effectiveness was verified. Then, lentiviral transduction was performed to express CAS9 and sgRNAs in Huh7 cells according to a previously described method, and antibiotic selection was performed (Concordet & Haeussler, 2018; Lin et al., 2022). A single‐cell clone was picked through limited dilution, and the knockout of *ZBTB7B* was confirmed through genotyping of the cellular DNA. AML12 and Huh7 cells were treated with vehicle (DMSO, Sangon Biotech, A600163‐0250), T0901317 (5 μM) (Selleck, S7076) or GW7647 (1 μM) (Sigma, G6793) for 24 h. The cell culture experiments were performed in triplicate and repeated at least three times.

### 2.8 Isolation and culture of primary hepatocytes

Primary hepatocytes were isolated via a two‐step collagenase perfusion method. After the mice were euthanized, the livers were perfused with perfusion buffer and collagenase successively through the right atrium. The moderately digested liver was minced, centrifuged and washed twice, and then the cells were spread in a culture dish coated with collagen. The cells were cultured in DMEM supplemented with 10% fetal bovine serum and 1% penicillin streptomycin in a humidified chamber at 37°C under a 5% CO_2_ atmosphere.

### 2.9 Metabolic analyses

Triglyceride (TG), nonesterified fatty acid (NEFA), cholesterol, ALT and AST levels were quantified via assay kits from Sigma (TG, TR0100), Wako (NEFA, 294‐63601), and Stanbio (ALT, 2930430, AST, 2920430 and cholesterol, 1010‐430) according to the manufacturers' protocols. TG and cholesterol in the liver were extracted via organic solvents, and their levels were measured via assay kits from Sigma (TG, TR0100) and Stanbio (cholesterol, 1010‐430) according to the manufacturers' protocols.

### 2.10 Cell counting Kit‐8 (CCK‐8) assay

AML12, Huh7 and HepG2 cells (2000 cells/well) were cultured in 96‐well plates. After attachment, working solution containing 10 μL of CCK8 (Selleck, B34304) reagent was added to each well at specific time points (0, 24, 48, 72 and 96 h), and the samples were incubated for 1 or 2 h. The absorbance at 450 nm was measured with a UV/Vis microplate spectrophotometer (Multiskan™ Sky Spectrophotometer, Thermo Scientific).

### 2.11 Colony formation assay

All the cells (1000 cells/well) were cultured in 6‐well plates supplemented with 2 mL of DMEM supplemented with 10% FBS and 1% PS for approximately 2 weeks until visible clones formed. The cells were fixed with 4% paraformaldehyde for 1 h and stained with 1% crystal violet solution (Biosharp, BS941) for 40 min. The number of colonies was quantitated by ImageJ software (National Institutes of Health, Bethesda, MD).

### 2.12 Wound healing assay

A total of 1.2 × 10^6^ cells were plated in 6‐well plates and incubated to reach 95%–100% confluence. Scratches were created via 200 μL pipettes, and the medium was replaced with DMEM containing 1% FBS and 1% PS after washing with PBS. Pictures were taken at 0 and 72 h, and the area of wound migration was calculated by ImageJ software.

### 2.13 Gene expression analyses

TRIzol reagent (Vazyme, R401‐01) was used to extract total RNA, HiScript II Q RT SuperMix was used for qPCR to synthesize cDNA (Vazyme, R222‐01), and Taq Pro Universal SYBR qPCR Master Mix (Vazyme, Q712‐02) was used to perform qPCR following the manufacturer's instructions. The primers used for amplification of the coding sequences are listed in Table S1.

### 2.14 Immunoblotting analysis

The liver cell lysates were prepared via lysis buffer containing 50 mM Tris–HCl (pH = 7.8), 137 mM NaCl, 10 mM NaF, 1 mM EDTA, 1% Triton X‐100, 10% glycerol, and a protease inhibitor cocktail (Bimake, B14002), followed by three freeze/thaw cycles. Thirty micrograms of each protein sample were separated via 8% SDS–PAGE and transferred to PVDF membranes (GE, 10600023). Then, the membranes were blocked with 5% nonfat milk (Beyotime, P0216‐300 g) at room temperature for 1.5 h and incubated with primary antibodies overnight at 4°C. The antibodies used were against HSP90 (Proteintech,13171‐1‐AP) and ZBTB7B (TH‐POK) (Santa Cruz, sc‐376250). Next, the membranes were incubated with either goat anti‐mouse IgG (H + L) (Jackson ImmunoResearch, 115‐035‐003) or goat anti‐rabbit IgG (H + L) (Jackson ImmunoResearch, 111‐035‐003) horseradish peroxidase‐conjugated polyclonal secondary antibodies at room temperature for 1.5 h. Proteins were visualized via Lumi Q reagent solution (Share Bio, SB‐WB012).

### 2.15 RNA‐seq data analysis

The fastq files were subjected to quality control via FastQC followed by alignment against the mouse reference genome (mm38) via the aligner HISAT2. HTSeq was used to count the number of reads mapped to each reference gene. DESeq2 was used for differential expression analysis. Transcripts exhibiting significant differences of more than 1.5‐fold in expression in the ZLKO group compared with the WT group were selected for further analysis.

### 2.16 Statistical analysis

Statistical analyses were conducted via GraphPad Prism 9. Two‐tailed unpaired Student's *t*‐tests were used to evaluate significant differences between two groups. Analysis of variance (ANOVA) and post hoc analyses were used to compare differences among multiple groups. The results are presented as the mean ± SD. Statistical methods and corresponding *p*‐values for the data shown in each panel are included in the figures and figure legends.

## 3 RESULTS

### 3.1 Zbtb7b knockout promotes lipid deposition in the liver upon HFD feeding

**FIGURE 1:**
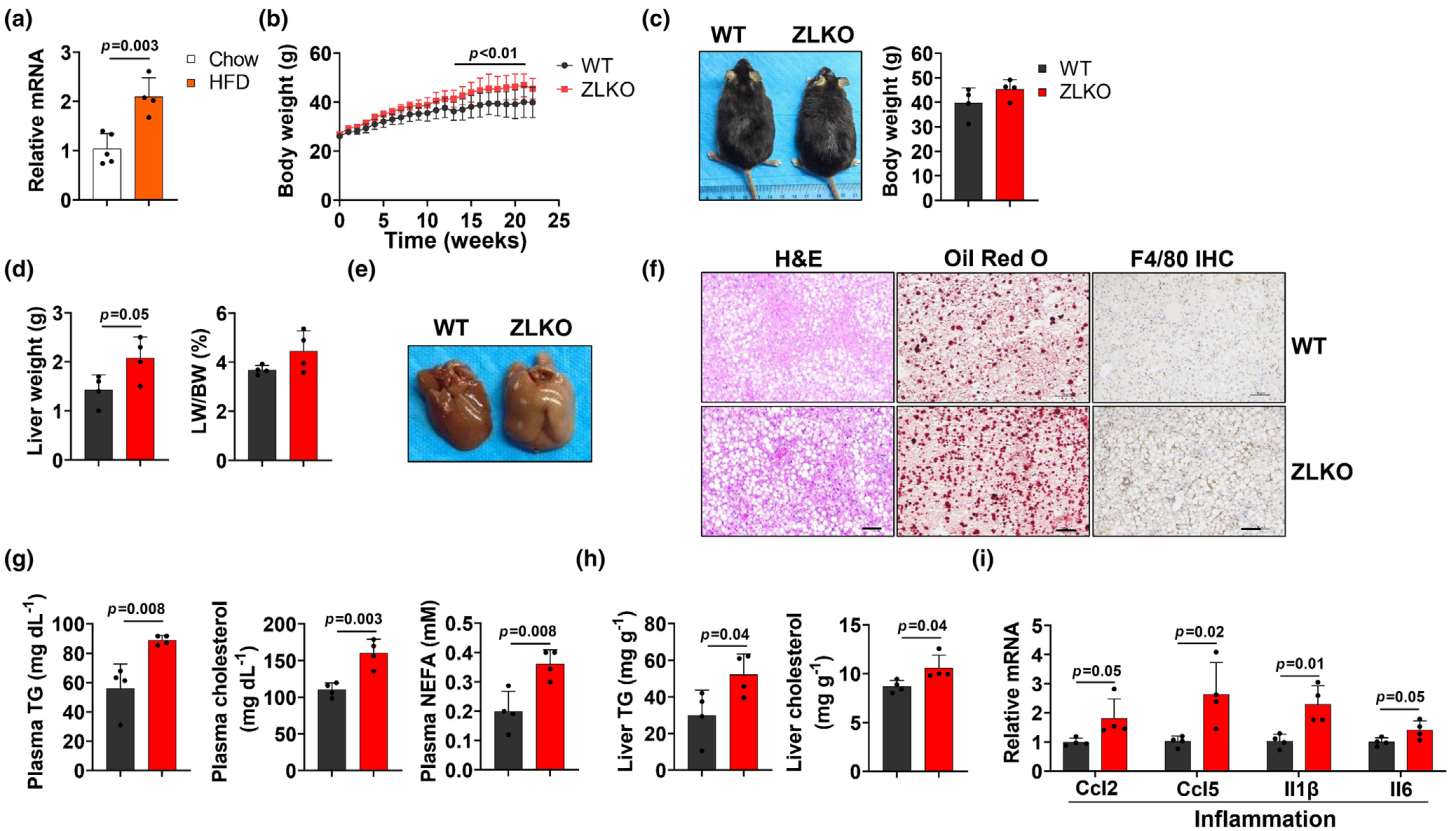
Zbtb7b knockout leads to lipid accumulation and inflammation in the liver upon HFD feeding. (a) QPCR analysis of the expression of Zbtb7b in the livers of wild type mice fed a chow diet (Chow, open, *n* = 5) or a high‐fat diet (HFD, filled, *n* = 4) for 4 months. (b) Changes in the body weights of AAV‐TBG‐control (WT, black, *n* = 4)‐ and AAV‐TBG‐Cre‐injected Zbtb7b^flox/flox^ (ZLKO, red, *n* = 4) mice fed high‐fat diet (HFD) for 5 months. (c) General images and body weights of WT and ZLKO mice after 5 months of HFD feeding. (d) Column plots of the liver weights (left) and liver‐to‐body weight ratios (right) of WT and ZLKO mice. (e) General images of the livers of WT and ZLKO mice after HFD feeding. (f) Hematoxylin and eosin (H&E) staining, ORO staining and F4/80 immunohistochemical (IHC) staining (scale bar = 100 μm) of livers from WT and ZLKO mice. (g) Plasma triglyceride (TG, left), cholesterol (middle) and nonesterified fatty acid (NEFA, right) levels in WT and ZLKO mice after HFD feeding. (h) Liver triglyceride (TG, left) and cholesterol (right) contents in WT and ZLKO mice. (i)The expression levels of hepatic genes involved in inflammation in WT and ZLKO mice after HFD feeding for 5 months. The data in a, c, d, and g–i are presented as the mean ± SD. WT versus ZLKO, two‐tailed unpaired Student's *t*‐test. The data in B represent the mean ± SD. WT versus ZLKO, two‐way ANOVA with multiple comparisons.

Zbtb7b is a transcriptional factor that potentially regulates lipid metabolism in the liver. We found that Zbtb7b expression was increased in a high‐fat diet (HFD)‐induced MASLD mouse model (Figure 1a). To explore the function of Zbtb7b in hepatic lipid homeostasis, we administered an AAV virus expressing Cre recombinase under the thyroxine‐binding globulin (TBG) promoter to Zbtb7b^flox/flox^ mice, thus specifically knocked out Zbtb7b in the liver (ZLKO) (Figure S1a). We observed no significant difference in body weight between ZLKO mice and control virus‐injected mice (WT), but the liver weights of ZLKO mice are heavier after chow diet feeding (Figure S1b–d). The liver morphology and histology were not obviously different between WT and ZLKO mice (Figure S1e). The plasma triglyceride (TG) did not differ, whereas the NEFA and cholesterol levels markedly increased (Figure S1f). To examine the role of Zbtb7b during MASLD development, we fed ZLKO and WT mice on HFD. During HFD feeding, the ZLKO mice gained more body weight than WT mice (Figure 1b). Upon being harvested, the body weights of the ZLKO mice were slightly heavier, but the liver weights of these mice were significantly heavier compared to WT mice (Figure 1c,d). Moreover, the livers of ZLKO mice appeared more steatotic and contained more lipid droplets, as shown by H&E staining and ORO staining. Immunohistochemical staining revealed increased macrophage infiltration in the livers of ZLKO mice (Figure 1e,f). Additionally, the levels of triglycerides, cholesterol and free fatty acids in the plasma and triglycerides and cholesterol in the livers of ZLKO mice were relatively higher (Figure 1g,h), suggesting that more lipids accumulated in the livers of ZLKO mice. Lipid deposition in the liver is a high risk factor for hepatic inflammation. We revealed that the expression of the inflammatory cytokines Ccl2, Ccl5, and Il1β was strongly induced in the livers of ZLKO mice after HFD feeding, which was closely associated with increased recruitment of macrophages to the liver (Figure 1i). Taken together, these results suggest that the hepatic knockout of Zbtb7b leads to lipid accumulation in the liver and triggers inflammation during HFD‐induced MASLD progression.

### 3.2 Knockout of Zbtb7b promotes the formation of MASLD‐related HCC

**FIGURE 2:**
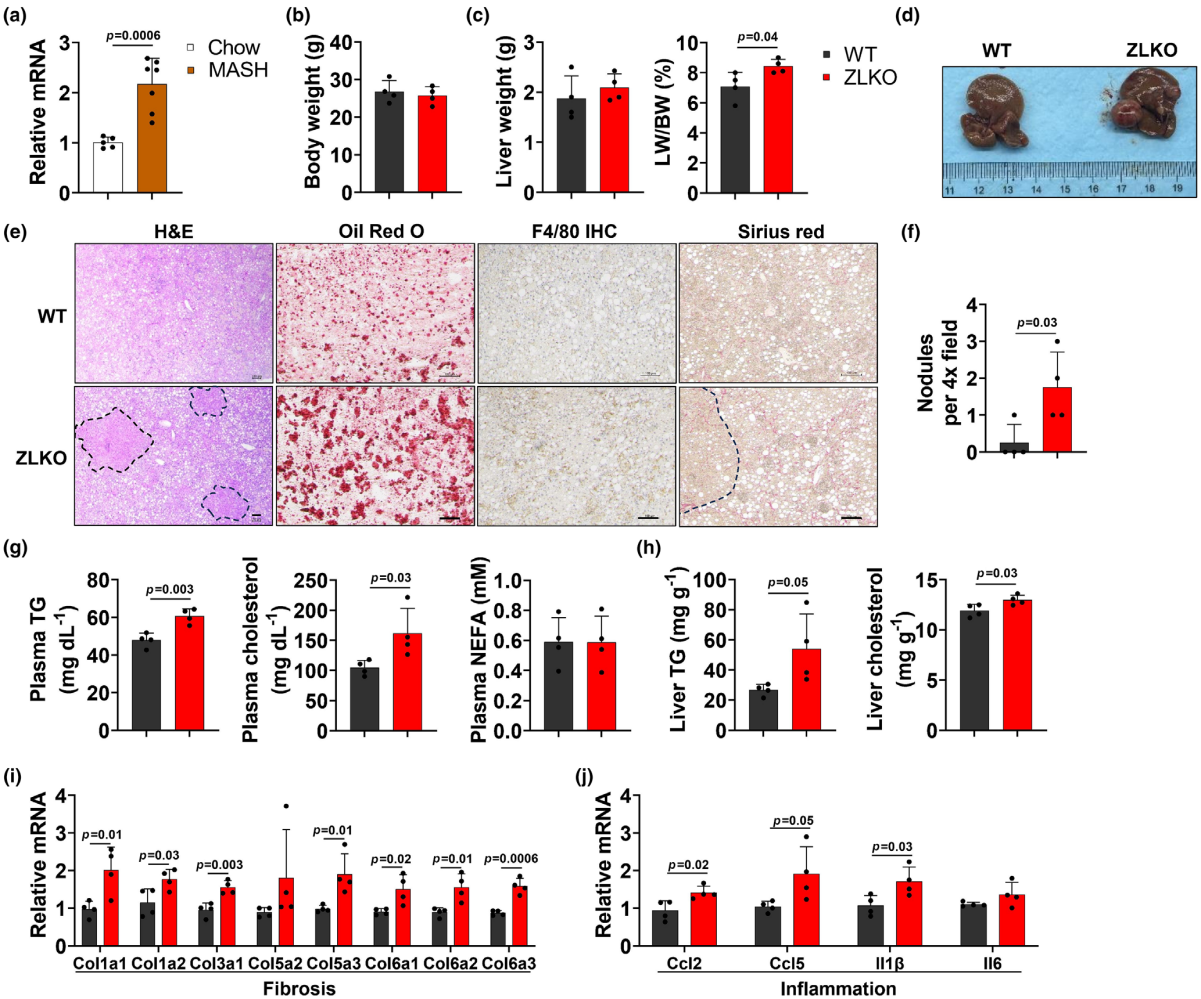
Zbtb7b knockout promotes MASLD‐related HCC progression. (a) QPCR analysis of the expression of Zbtb7b in the livers of wild type mice after chow diet (Chow, open, *n* = 5) or CDA‐HFD diet (MASH, filled, *n* = 7) feeding for 4 months. (b) Body weights of AAV‐TBG‐control (WT, black, *n* = 4) and AAV‐TBG‐Cre‐injected Zbtb7b^flox/flox^ (ZLKO, red, *n* = 4) mice after MASH diet feeding for 7 months. (c) Liver weights (left) and liver‐to‐body weight ratios (right) of WT and ZLKO mice. (d) Representative liver images of WT and ZLKO mice after feeding MASH diet for 7 months. (e) Hematoxylin and eosin (H&E) staining, ORO staining, F4/80 immunohistochemical (IHC) staining and Sirius red staining (scale bar = 100 μm) of livers from WT and ZLKO mice after MASH diet feeding. (f) Column plot of the number of nodules per 4× field in WT and ZLKO mice. (g) Plasma triglyceride (TG, left), cholesterol (middle) and nonesterified fatty acid (NEFA, right) levels of WT and ZLKO mice after MASH diet feeding. (h) Liver TG (left) and cholesterol (right) contents in WT and ZLKO mice. (i, j) The expression of genes associated with inflammation and fibrosis in the livers of WT and ZLKO mice after MASH diet feeding for 7 months. The data in a–c and f–j are presented as the mean ± SD. WT versus ZLKO, two‐tailed unpaired Student's *t*‐test.

Some patients with MASLD progress to HCC. Next, we investigated whether Zbtb7b deficiency influences the MASLD‐to‐HCC transition by feeding mice on CDA‐HFD (MASH diet), a commonly used metabolic dysfunction‐associated steatohepatitis (MASH) model, for 7 months. The expression of Zbtb7b in the liver of WT mice was elevated after MASH diet feeding (Figure 2a). Notably, the liver weights of the ZLKO mice increased, which was consistent with the HFD results, although the body weights of these mice did not differ (Figure 2b,c). In addition, ZLKO mice presented increased lipid accumulation, macrophage infiltration and collagen deposition in the liver, and more interestingly, ZLKO mice started to develop tumors in the liver with the occurrence of tumor nodules, as shown by general liver images, H&E staining, ORO staining, Immunohistochemical staining and Sirius red staining of the liver (Figure 2d–f). The plasma triglyceride and cholesterol levels were augmented in the ZLKO mice, whereas the plasma NEFA levels were unchanged (Figure 2g). Additionally, triglyceride and cholesterol levels in the liver were also elevated in ZLKO mice (Figure 2h). Furthermore, we revealed that inflammation and fibrosis‐related gene expression was substantially elevated in ZLKO mice after MASH diet feeding (Figure 2i,j), indicating that lipogenesis, inflammation and fibrosis, all these MASH phenotypes are induced in ZLKO mice along with the initiation of HCC. To further confirm that hepatic Zbtb7b regulates HCC development, we generated an Akt/N‐Ras‐induced HCC model to assess the role of Zbtb7b in modulating oncogene‐driven HCC progression. ZLKO and WT mice presented no difference in body weight, whereas liver weights notably increased in ZLKO mice after HCC induction (Figure S2a,b). Consistent with the findings in the MASLD‐induced HCC model, Zbtb7b knockout also facilitated oncogene‐induced HCC progression. The number of tumor nodules in the liver was significantly more in ZLKO mice than in WT mice while enhancive hepatic steatosis was observed in ZLKO mice (Figure S2c,d). In addition, the contents of ALT and AST in the plasma were elevated, indicating greater liver damage in ZLKO mice than in WT mice (Figure S2e). Furthermore, the plasma NEFA, TG, cholesterol and hepatic triglyceride, and cholesterol levels were significantly increased in the Akt/N‐Ras‐induced HCC model (Figure S2f–g), suggesting that increased lipid accumulation in the livers of Zbtb7b knockout mice may also promote HCC progression in an oncogene‐induced HCC model. In summary, these results suggest that Zbtb7b knockout promotes hepatic lipid accumulation, inflammation and fibrosis in a MASH condition, which may facilitate the MASLD‐to‐HCC transition and further HCC progression.

### 3.3 Zbtb7b overexpression suppresses hepatic cell proliferation and migration

**FIGURE 3:**
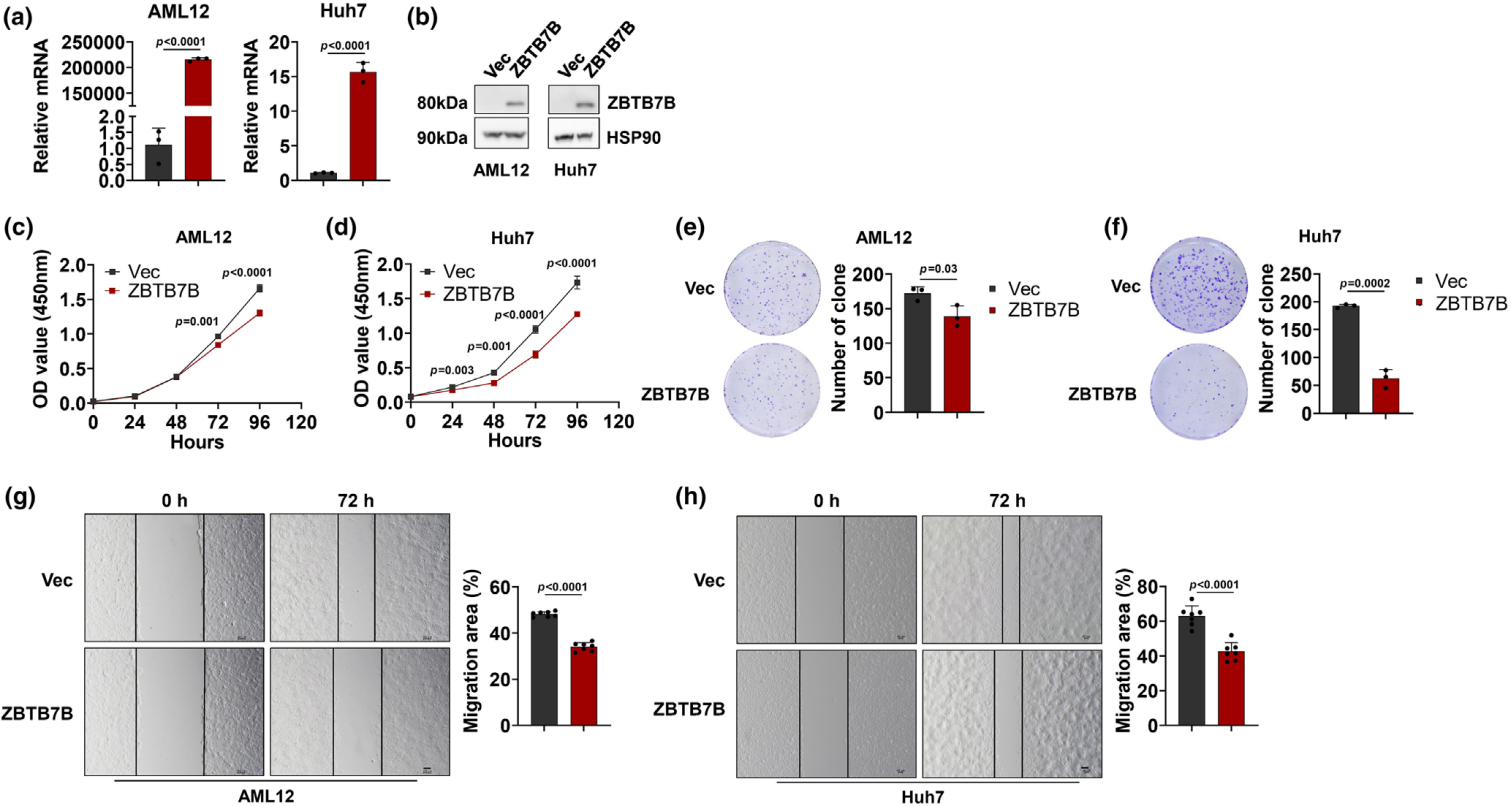
Zbtb7b inhibits the proliferation and migration of hepatic cells. (a) QPCR analysis of the expression of ZBTB7B in Vec‐ (black, *n* = 3) and ZBTB7B‐overexpressing (claret, *n* = 3) AML12 and Huh7 cells. (b) Immunoblotting of lysates from vector (Vec) and ZBTB7B‐overexpressing (ZBTB7B) AML12 (left) and Huh7 cells (right). (c, d) The proliferation ability of Vec‐ and ZBTB7B‐overexpressing AML12 cells (c) and Huh7 cells (d) was detected via Cell Counting Kit‐8 (CCK‐8). (e, f) The colony formation ability of Vec‐ and ZBTB7B‐overexpressing AML12 cells (e) and Huh7 cells (f) was detected via colony formation assays. (g, h) Wound healing assay for Vec‐ and ZBTB7B‐overexpressing AML12 cells (g) and Huh7 cells (h). The wound sizes (*n* = 7) were measured at 0 h and 72 h after scratching. Scale bar = 100 μm. The data in a, e–h are presented as the mean ± SD. Vec versus ZBTB7B, two‐tailed unpaired Student's *t*‐test. The data in c, d represent the mean ± SD. Vec versus ZBTB7B, two‐way ANOVA with multiple comparisons.

Loss of Zbtb7b promotes MASLD‐to‐HCC progression. To determine whether Zbtb7b expression cell‐autonomously mediates hepatic cell proliferation and migration under both premalignant and malignant conditions, we used the normal hepatocyte cell line AML12 and the HCC cell lines Huh7 and HepG2 to establish stable ZBTB7B‐overexpressing cell lines. ZBTB7B‐overexpressing AML12, Huh7 and HepG2 cells highly expressed Zbtb7b mRNA and protein (Figures 3a,b and S3a,b). Then, we performed CCK8 and colony formation assays in these cell lines to assess the proliferation abilities of cells after ZBTB7B overexpression. The results revealed that the overexpression of ZBTB7B significantly inhibited proliferation and substantially reduced the colony formation abilities of these cells (Figures 3c–f and S3c,d). Additionally, scratch assays demonstrated that ZBTB7B overexpression suppressed the migration ability of AML12 and Huh7 cells (Figure 3g,h). Overall, these results provide evidence that ZBTB7B expression suppresses both normal hepatocyte and HCC cells proliferation and migration and therefore may impede HCC formation and progression.

### 3.4 Knockout of Zbtb7b promotes LXR‐Srebp1c‐mediated de novo lipogenesis and inhibits PPARα‐induced fatty acid oxidation in the liver

**FIGURE 4:**
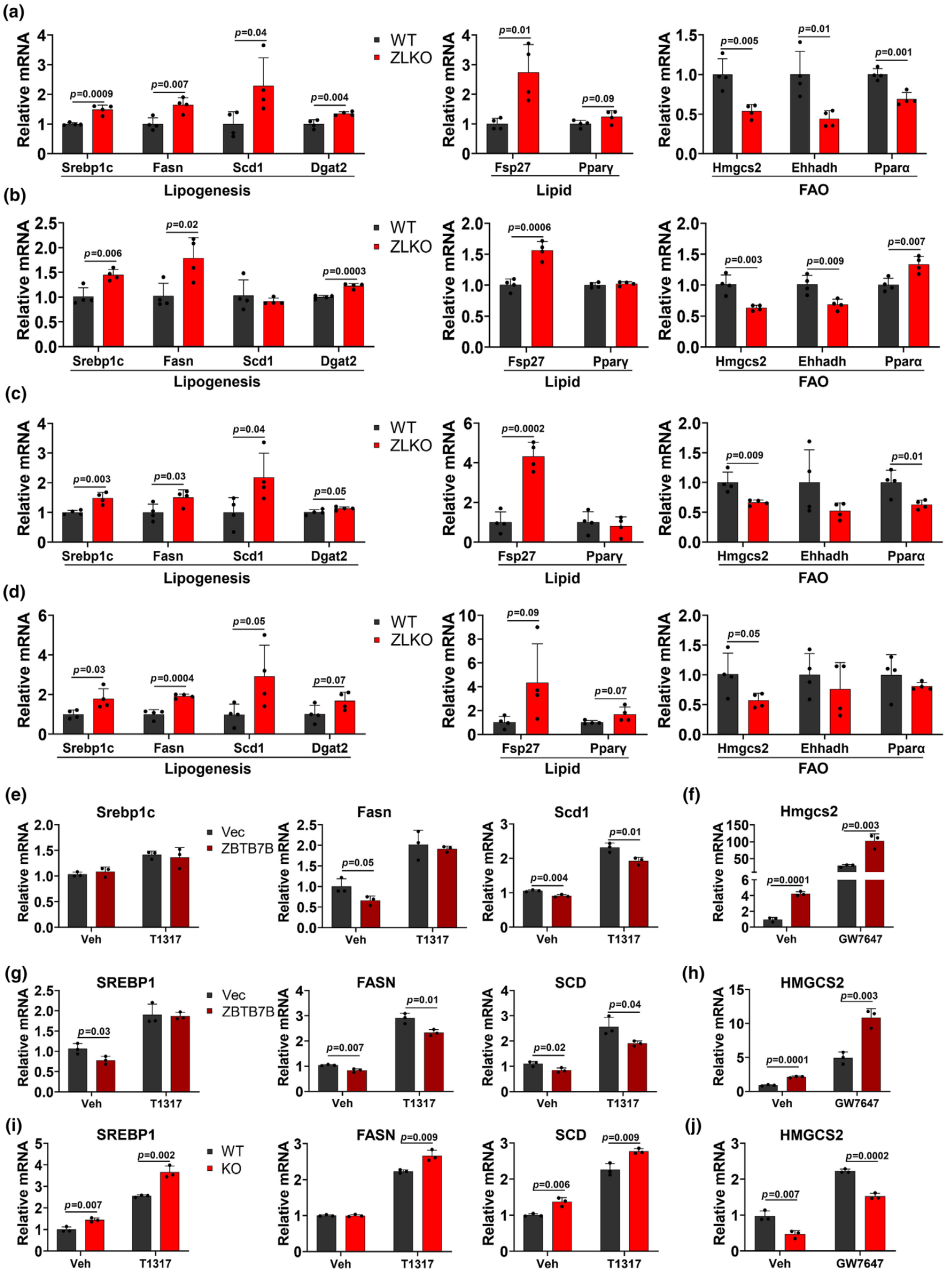
Deficiency of Zbtb7b increases de novo lipogenesis and decreases fatty acid oxidation‐related gene expression in hepatocytes. (a) QPCR analysis of hepatic genes involved in lipogenesis, lipid information (Lipid) and fatty acid oxidation (FAO) in AAV‐TBG‐control (WT, black, *n* = 4) and AAV‐TBG‐Cre‐injected Zbtb7b^flox/flox^ (ZLKO, red, *n* = 4) mice after chow diet feeding for 1 week. (b) Relative mRNA expression of hepatic genes involved in lipid metabolism in primary hepatocytes isolated from WT (black, *n* = 4) and ZLKO mice (red, *n* = 4) after chow diet feeding for 1 week was determined by qPCR. (c, d) The expression of genes associated with lipid metabolism in the livers of WT (black, *n* = 4) and ZLKO mice (red, *n* = 4) after HFD feeding for 5 months (c) or MASH diet feeding for 7 months (d) was measured via qPCR. (e) QPCR analysis of lipogenic genes in control (Vec, black, *n* = 3) and ZBTB7B‐overexpressing (ZBTB7B, claret, *n* = 3) AML12 cells treated with vehicle (Veh) or T0901317 (T1317, 5 μM) for 24 h. (f) QPCR analysis of Hmgcs2 expression in Vec‐ (black, *n* = 3) and ZBTB7B‐overexpressing AML12 cells (claret, *n* = 3) treated with vehicle (Veh) or GW7647 (1 μM) for 24 h. (g) QPCR analysis of lipogenic genes in Vec‐ (black, *n* = 3) and ZBTB7B‐overexpressing Huh7 cells (claret, *n* = 3) treated with vehicle (Veh) or T0901317 (T1317, 5 μM) for 24 h. (h) QPCR analysis of HMGCS2 expression in Vec‐ (black, *n* = 3) and ZBTB7B‐overexpressing Huh7 cells (claret, *n* = 3) treated with vehicle (Veh) or GW7647 (1 μM) for 24 h. (i) QPCR analysis of lipogenic genes in wild‐type (WT, black, *n* = 3) and Zbtb7b sgRNA‐containing lentivirus‐transduced CAS9‐expressing Huh7 cells (KO, red, *n* = 3) treated with vehicle (Veh) or T0901317 (T1317, 5 μM) for 24 h. (j) QPCR analysis of HMGCS2 expression in WT (black, *n* = 3) and KO cells (red, *n* = 3) treated with vehicle (Veh) or GW7647 (1 μM) for 24 h. The data in a–j are presented as the mean ± SD. a–d, WT versus ZLKO, e–h, Vec versus ZBTB7B, i–j, WT versus KO, two‐tailed unpaired Student's *t*‐test.

The increase in hepatic steatosis in ZLKO mice is strongly associated with HCC development. The aberrantly increased de novo lipogenesis and compromised fatty acid oxidation in the liver are signatures of MASLD progression and may serve as pathogenic factors for the MASLD‐to‐HCC transition. Thus, we further measured the expression of de novo lipogenesis genes such as Srebp1c, Fasn, Scd1, and Dgat2 and the lipid formation markers Fsp27 and Pparγ. And the fatty acid oxidation genes Hmgcs2, Ehhadh, and Pparα in the livers of WT and ZLKO mice fed a normal diet. The results revealed that the expression of genes associated with de novo lipogenesis and lipid formation was significantly upregulated, whereas the expression of genes associated with fatty acid oxidation was markedly downregulated in the livers of ZLKO mice (Figure 4a). Importantly, this effect is cell autonomous, which is supported by the induction of de novo lipogenesis genes and the suppression of fatty acid oxidation genes in primary hepatocytes isolated from WT and ZLKO mice (Figure 4b). Notably, the induction of de novo lipogenesis genes and lipid formation genes and the inhibition of fatty acid oxidation genes were consistently observed in the livers of ZLKO mice after HFD feeding, MASH diet feeding and oncogene‐driven HCC progression (Figures 4c,d and S2h–j), indicating that ZBTB7B expression in the liver is required for the suppression of de novo lipogenesis and activation of fatty acid oxidation in the liver, which are considered protective mechanisms for MASLD development and HCC progression. Interestingly, Zbtb7b deficiency suppressed Ldlr and induced Pcsk9 expression, which mediates hepatic cholesterol uptake, thereby increasing plasma cholesterol levels. Additionally, the expression of Abcg5 and Abcg8, which are involved in the efflux of cholesterol in hepatocytes, was attenuated upon Zbtb7b depletion, although the expression of cholesterol synthesis‐related genes was unchanged (Figure S4). The dysregulation of these genes by Zbtb7b deficiency may lead to cholesterol accumulation in the liver.

Key transcriptional factors, such as Srebp1c and Pparα, mediate hepatic de novo lipogenesis and fatty acid oxidation programs, respectively. To evaluate whether ZBTB7B expression influences SREBP1c‐ and PPARα‐mediated de novo lipogenesis and the fatty acid oxidation pathway, we further treated ZBTB7B‐overexpressing or Zbtb7b‐depleted AML12 and Huh7 cells with T0901317, an agonist of the liver X receptor (LXR)‐SREBP1c pathway that induces de novo lipogenesis, and GW7647, a ligand for PPARα, which increases hepatic β‐oxidation, to assess the expression of de novo lipogenic genes, lipid formation genes and fatty acid oxidation genes. The results showed that the overexpression of ZBTB7B inhibited LXR‐SREBP1c pathway activation‐induced lipogenic genes such as Srebp1c, Fasn, and Scd1 and promoted PPARα‐induced fatty acid oxidation genes, such as Hmgcs2, in the AML12, and Huh7 cell lines (Figure 4e–h). In contrast, the knockout of Zbtb7b in Huh7 cells promoted the expression of de novo lipid synthesis genes and inhibited the expression of fatty acid oxidation genes (Figure 4i,j). These results indicated that ZBTB7B expression may regulate de novo lipogenesis and fatty acid oxidation by modulating LXR‐SREBP1c‐ and PPARα‐mediated programs.

### 3.5 Knockout of Zbtb7b upregulates H19 expression

**FIGURE 5:**
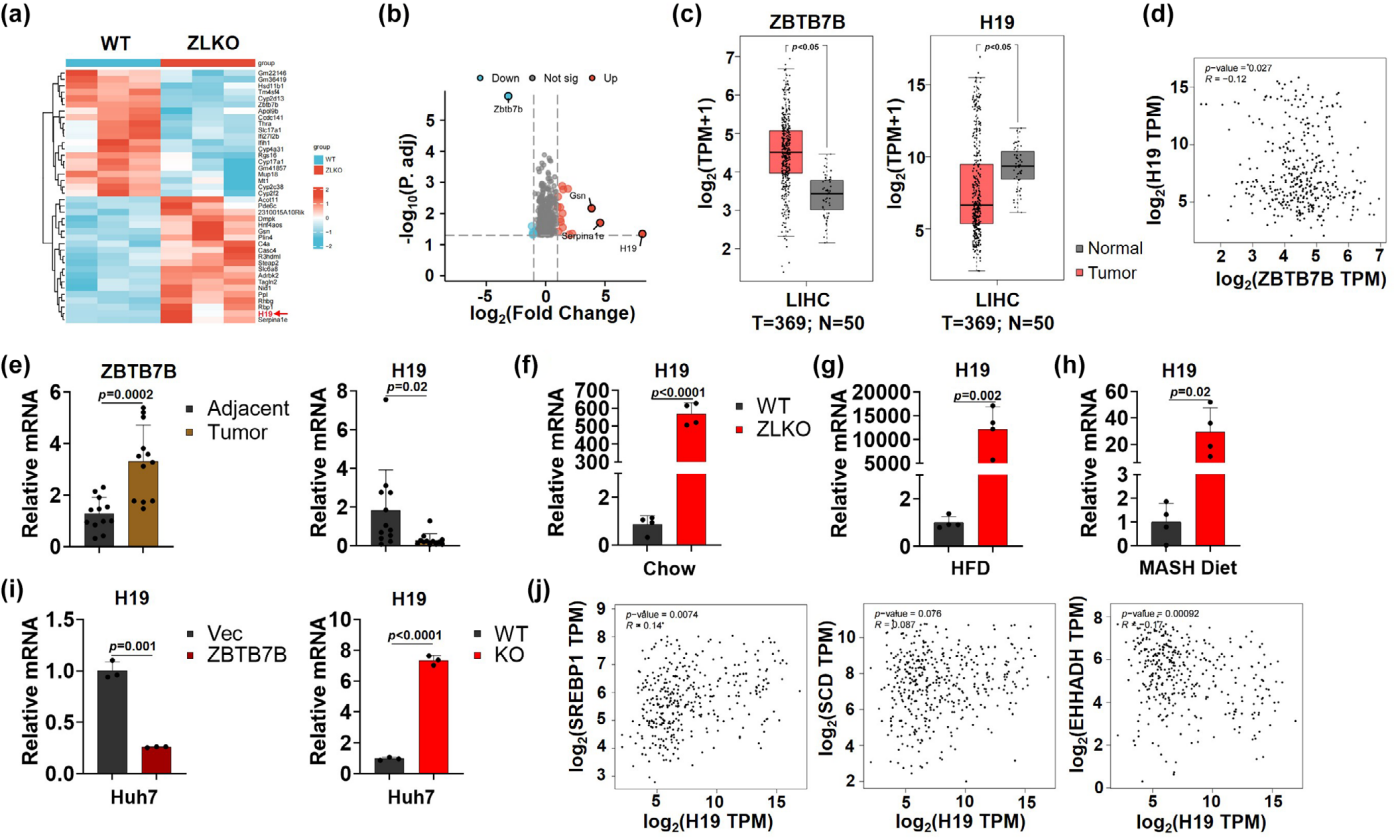
ZBTB7B knockout induces H19 expression. (a) Heatmap showed the differentially expressed genes identified by RNA sequencing in the livers of AAV‐TBG‐control (WT, left, *n* = 3)‐ and AAV‐TBG‐Cre‐injected Zbtb7b^flox/flox^ (ZLKO, right, *n* = 3) mice fed a chow diet for 1 week. (b) Volcano plot presented the upregulated (red) and downregulated (blue) genes in the livers of AAV‐TBG‐Cre‐injected Zbtb7bflox/flox (ZLKO) mice compared with those in the livers of AAV‐TBG‐control‐injected (WT) mice. (c) GEPIA (https://gepia.cancer‐pku.cn/) was used to determine the expression profiles of ZBTB7B and H19 in human HCC tissues (red) and normal tissues (gray). (d) Scatter plot obtained from GEPIA showing the negative correlation between ZBTB7B and H19 in human HCC tissues. (e) QPCR analysis of the expression of ZBTB7B and H19 in human HCC tissues (yellow, *n* = 12) and adjacent tissues (black. *n* = 12). (f–h) QPCR analysis of H19 expression in the livers of WT (black, *n* = 4) and ZLKO mice (red, *n* = 4) after chow diet feeding for 1 week (f), HFD feeding for 5 months (g) and MASH diet feeding for 7 months (h). (i) H19 expression in vector (Vec, black, *n* = 3) and ZBTB7B‐overexpressing (ZBTB7B, claret, *n* = 3) or wild‐type (WT, black, *n* = 3) and Zbtb7b‐knockout (KO, red, *n* = 3) Huh7 cells was measured via qPCR. (j) Scatter plots obtained from GEPIA showed the correlations between H19 and SREBP1, SCD or EHHADH in human HCC tissues. The data in c, e–i are presented as the mean ± SD. C, Normal versus Tumor, E, Adjacent versus Tumor, f–h, WT versus ZLKO, i, Vec versus ZBTB7B (left), WT versus KO (right), two‐tailed unpaired Student's *t*‐test.

The above results indicate that ZBTB7B exacerbates the development of HCC by mediating LXR‐SREBP1c‐ and PPARα‐associated hepatic de novo lipogenesis and fatty acid oxidation programs. However, the molecular mechanism mediated by ZBTB7B expression remains unclear. Therefore, we performed RNA sequencing on liver RNA from WT and ZLKO mice. Heatmap and volcano plot showing the significantly upregulated and downregulated genes in the ZLKO mouse livers. Among them, H19 was the most highly upregulated gene upon Zbtb7b depletion (Figure 5a,b). In an HCC dataset from the TCGA database, we observed increased ZBTB7B expression and decreased H19 expression in HCC patients compared with healthy individuals (Figure 5c). Interestingly, we also noticed a negative correlation between ZBTB7B and H19 expression in the TCGA database and a group of human HCC samples (Figure 5d,e and Table S2). Furthermore, we verified that H19 was upregulated in the livers of ZLKO mice fed a chow diet, HFD, MASH diet and driven HCC by expressing oncogenes (Figures 5f–h and S2k). In addition, the overexpression of ZBTB7B in Huh7 cells downregulated H19 expression, whereas Zbtb7b‐knockout Huh7 cells upregulated H19 expression, indicating that Zbtb7b cell‐autonomously suppressed H19 expression (Figure 5i). H19 is the first lncRNA to be identified and defined as an imprinted gene with a conserved secondary structure (Hashemi et al., 2022). Increasing evidence suggests that H19 plays a crucial role in various human cancers, including HCC (Raveh et al., 2015). Importantly, we detected a significant positive correlation between H19 and the de novo lipogenesis genes SREBP1 and SCD, and a significant negative correlation with the fatty acid oxidation gene EHHADH in an HCC dataset from the TCGA database (Figure 5j). These results suggest that Zbtb7b suppresses H19 expression in the liver during MASLD progression and HCC development. The inhibition of H19 by Zbtb7b may play an important role in regulating hepatic de novo lipogenesis and fatty acid oxidation processes.

### 3.6 H19 overexpression offsets the protective effect of Zbtb7b on the proliferation and migration of hepatic cells

**FIGURE 6:**
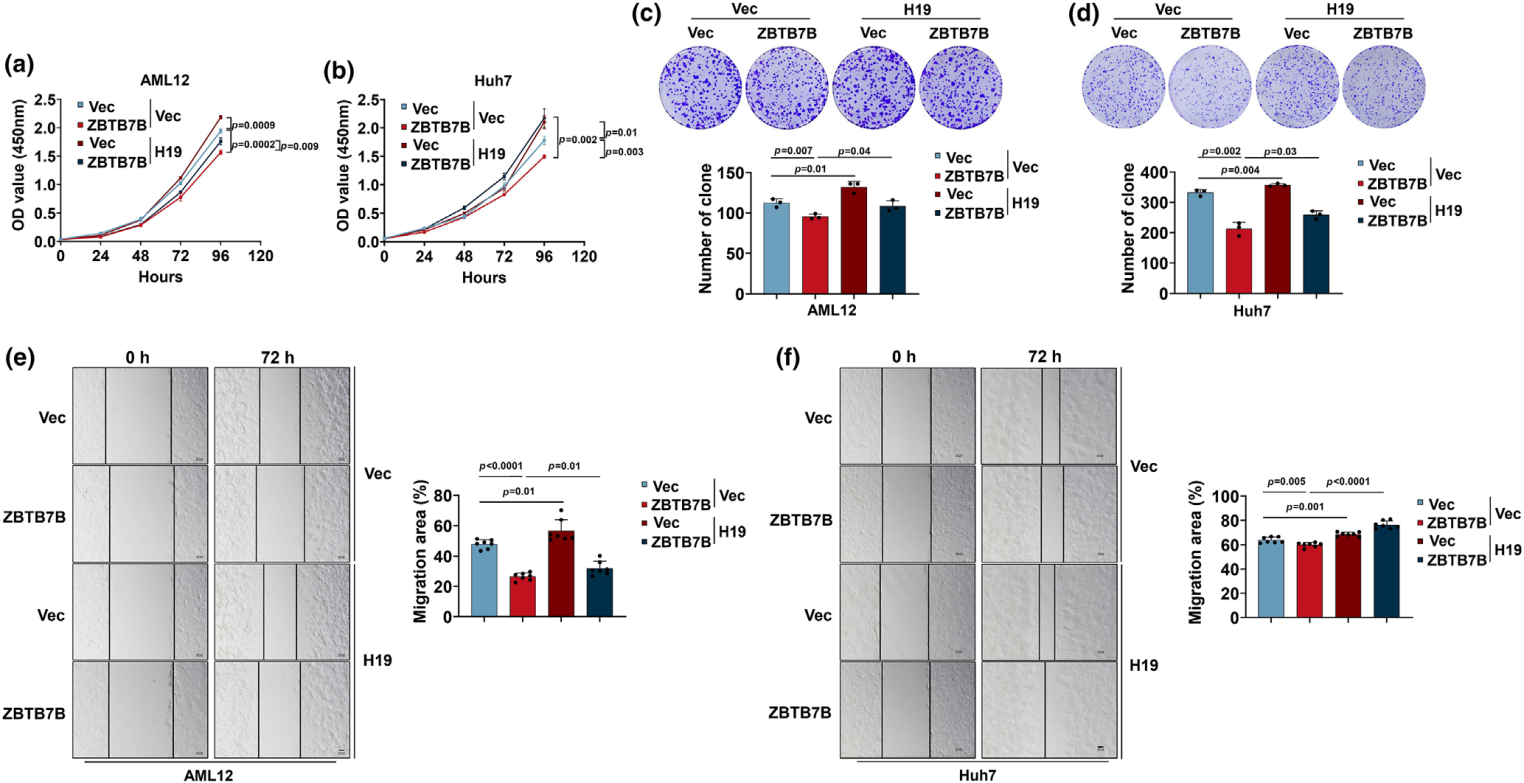
Zbtb7b overexpression inhibits hepatic cell proliferation and migration by suppressing H19 expression.(a–d) Vector (Vec) and H19 were stably transduced by retrovirus in vector (Vec, *n* = 3) and ZBTB7B‐overexpressing (ZBTB7B, *n* = 3) AML12 (a, c) and Huh7 (b, d) cells. The proliferation ability of the cells was detected via Cell Counting Kit‐8 (CCK‐8, a, b) and colony formation assays (c, d). (e, f) Vec and H19 were overexpressed in vector (Vec, *n* = 3)‐ and ZBTB7B‐overexpressing (ZBTB7B, *n* = 3) AML12 (e) and Huh7 (f) cells. The wound healing assays were performed with these cells, and the wound sizes (*n* = 7) were measured at 0 and 72 h after scratching. Scale bar = 100 μm. The data in A‐B represent the mean ± SD. Vec versus ZBTB7B, two‐way ANOVA with multiple comparisons. The data in c–f are presented as the mean ± SD., two‐tailed unpaired Student's *t*‐test.

To evaluate whether Zbtb7b protects against HCC progression by downregulating H19, we overexpressed H19 in ZBTB7B‐overexpressing AML12 and Huh7 cells. Consistent with previous results, in both AML12 and Huh7 cells, ZBTB7B overexpression suppressed cell proliferation, as measured by CCK8 assays and colony formation assays (Figure 6a–d), and cell migration, as measured by wound healing assays (Figure 6e,f). Intriguingly, H19‐expressing cells significantly enhanced cell proliferation and migration and attenuated the inhibitory effect induced by ZBTB7B expression in both AML12 cells and Huh7 cells. These results demonstrate that H19 is a key oncogenic factor that is suppressed by Zbtb7b in normal hepatocytes and HCC cells.

## 4 DISCUSSION

Metabolic dysfunction in the liver is tightly linked to liver diseases, including MASLD and HCC. MASLD is a spectrum of liver damage ranging from simple fatty liver to hepatic fibrosis and eventually to HCC. The overwhelming deposition of lipids in the liver, which consists of triglycerides, causes steatosis and is a diagnostic marker of MASLD progression. The amount of hepatic triglycerides is modulated by the de novo synthesis of fatty acids and their degradation by oxidation. To date, the aberrant synthesis of fatty acids is widely known as an important risk factor that drives MASLD progression. It is well documented that increased lipid accumulation in the liver is controlled by abnormally induced de novo lipogenesis via increased expression of the master regulator SREBP1c. On the other hand, the induction of fatty acid oxidation by PPARα, a key regulator of fatty acid oxidation, suppresses triglyceride formation and lipid accumulation in the liver. Therefore, a specific activator of PPARα has been developed as a potential therapeutic approach for MASLD (Monroy‐Ramirez et al., 2021, Pawlak et al., 2015). However, whether an endogenous compensatory mechanism occurs in the early stage of MASLD to reduce MASLD‐to‐HCC progression is not well understood. In this study, we demonstrated that the upregulated expression of Zbtb7b along with MASLD‐related HCC progression impedes the de novo lipogenesis process and enhances fatty acid oxidation. Zbtb7b depletion accelerated the MASLD process by increasing the plasma and liver TG and cholesterol contents and leading to hepatic steatosis and inflammation upon HFD feeding. Under diet‐induced MASH conditions, Zbtb7b deficiency directly drove HCC development. These findings suggest that Zbtb7b mediates a compensatory pathway that is critical for protecting the liver from MAFLD‐related HCC progression. Interestingly, loss of Zbtb7b further aggravated oncogene‐driven HCC development, indicating that metabolic dysregulation may also augment HCC cells malignancy. In support of this, the overexpression of ZBTB7B largely inhibited HCC cells proliferation and migration, probably by reducing de novo lipogenesis and increasing fatty acid oxidation to clear excess synthesized fatty acids in HCC cells. Currently, fatty acids are widely considered beyond serving as energy buffers to support tumor growth but function as signaling molecules that activate oncogenic pathways. Studies have shown that Zbtb7b knockout promotes oncogene‐induced tumor formation by activating the c‐Jun, Erk and NF‐κB pathways (Chen et al., 2022; Xia et al., 2021; Zhu et al., 2024), whereas our study revealed that fatty acid synthesis controlled by Zbtb7b plays a critical role in MASLD‐related HCC, probably in synergy with known Zbtb7b‐regulated pathways in driving tumor initiation and progression. Importantly, we demonstrated that Zbtb7b expression negatively regulates H19 expression. This result is supported by the negative correlation between Zbtb7b expression and H19 in mouse and human liver samples. H19 exerts its oncogenic effects via different mechanisms, which might depend on the type of cancer (Gamaev et al., 2021, Wang, Ye, et al., 2016, Yan et al., 2017). Notably, H19 expression augments de novo lipogenesis (Liu et al., 2018). A decrease in H19 suppresses hepatic de novo lipogenesis via the inhibition of PPARγ‐mediated SREBP1c activation (Liu et al., 2019). In this study, we revealed that the upregulation of H19 in hepatic Zbtb7b‐deficient mice may regulate both de novo lipogenesis and fatty acid oxidation programs. The overexpression of H19 in ZBTB7B‐overexpressing HCC cells attenuated the inhibitory effect of Zbtb7b by reactivating de novo lipogenesis and halting fatty acid oxidation, however, the detailed mechanisms by which Zbtb7b suppresses H19 expression need further investigation.

Overall, we revealed that Zbtb7b upregulation in the liver during MASLD to HCC progression functions as a defense mechanism. Zbtb7b mainly plays a role in decreasing fatty acid synthesis and increasing oxidation by H19, indicating that Zbtb7b overexpression could be a potential strategy for restricting MASLD‐related HCC development.

### 4.1 Limitations of the study

Most of our studies were performed at the transcriptional level, which limits the interpretation of gene function at the protein level. Although Zbtb7b and H19 expression was strongly correlated with the expression of lipogenesis‐ and fatty acid oxidation‐related genes, more evidence is needed to confirm the regulation of Zbtb7b on the expression of these genes via H19. The detailed mechanism also needs to be further investigated in future research.

## AUTHOR CONTRIBUTIONS

Y.H., H.J., and X.Y.Z. conceived the project and designed the research. Y.H. performed the majority of the studies. K.W., X.P., Y.F., and W.L. performed some animal and cell experiments. Y.H., J.M., H.J., and X.Y.Z. analyzed the data and wrote the manuscript.

## FUNDING INFORMATION

This work was supported by the National Key R&D Program of China (2023YFA1800802, 2020YFA0803603 to X.Y.Z.), the National Natural Science Foundation of China (82,070,894 to X.Y.Z.), and the Science and Technology Commission of Shanghai Municipality (22ZR1479800 to X.Y.Z.). Shanghai Frontiers Science Center of Cellular Homeostasis and Human Diseases. Innovative research team of high‐level local universities in Shanghai (SHSMU‐ZDCX20212501 to X.Y.Z.).

## CONFLICT OF INTEREST STATEMENT

The authors declare that they have no competing interests.

## ETHICS STATEMENT

The experimental protocol was approved by the University Committee on the Use and Care of Animals at Shanghai Jiao Tong University (Reference Number: A2022‐064). The research was prospectively reviewed and approved by the Ethics Committee of Zhongshan Hospital (Reference Number: 2022BAT6946), and informed consent was obtained from the participants. All the research was conducted in accordance with both the Declarations of Helsinki and Istanbul.

## Supporting information


Data S1:

## Data Availability

The RNA‐seq data files have been deposited in the Gene Expression Omnibus (www.ncbi.nlm.nih.gov/geo/) under accession number GSE277617. The authors confirm that the datasets used and analyzed during the current study are available from the corresponding author upon reasonable request.
