# Supplementary material for: Zbtb7b defines a compensatory mechanism in MASLD‐related HCC progression by suppressing H19‐mediated hepatic lipid deposition
Source: Physiol Rep. 2024 Dec 23;12(24):e70160. doi: 10.14814/phy2.70160 (PMC11664540; doi:10.14814/phy2.70160)
Supplement: Supplementary file 1 — Data S1: [file PHY2-12-e70160-s001.docx]

**Supplemental Information**

**FIGURES**


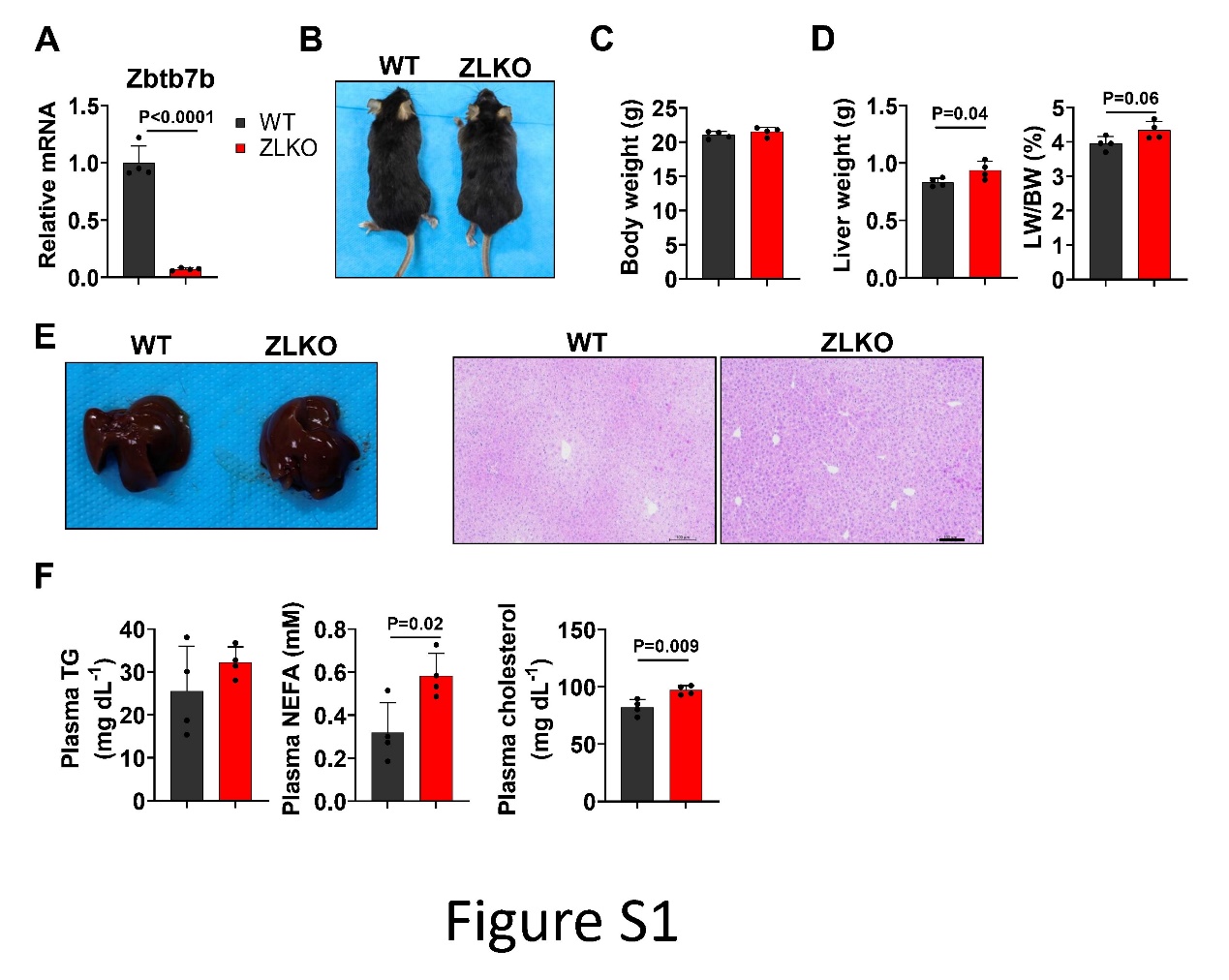


**Figure S1. Zbtb7b knockout regulates lipid metabolism in chow-diet fed mice.**

1. QPCR analysis of Zbtb7b expression in the liver of AAV-TBG-control (WT, black, n=4) and AAV-TBG-Cre-injected Zbtb7b^flox/flox^ (ZLKO, red, n=4) mice fed chow diet for one week.
2. General images of WT and ZLKO mice fed chow diet for one week.
3. The body weights of WT and ZLKO mice after one week chow-diet feeding.
4. Column plots of the liver weights (left) and liver-to-body weight ratios (right) of WT and ZLKO mice after chow-diet feeding.
5. General images of the livers and hematoxylin and eosin (H&E) staining (scale bar=100 μm) of livers of WT and ZLKO mice after chow-diet feeding for one week.
6. Column plots of the plasma triglyceride (TG, left), non-esterified fatty acid (NEFA, middle), and cholesterol (right) contents of WT and ZLKO mice.

The data in A, C-D and F represent as mean ± SD. WT vs. ZLKO, two-tailed unpaired Student’s t-tests.


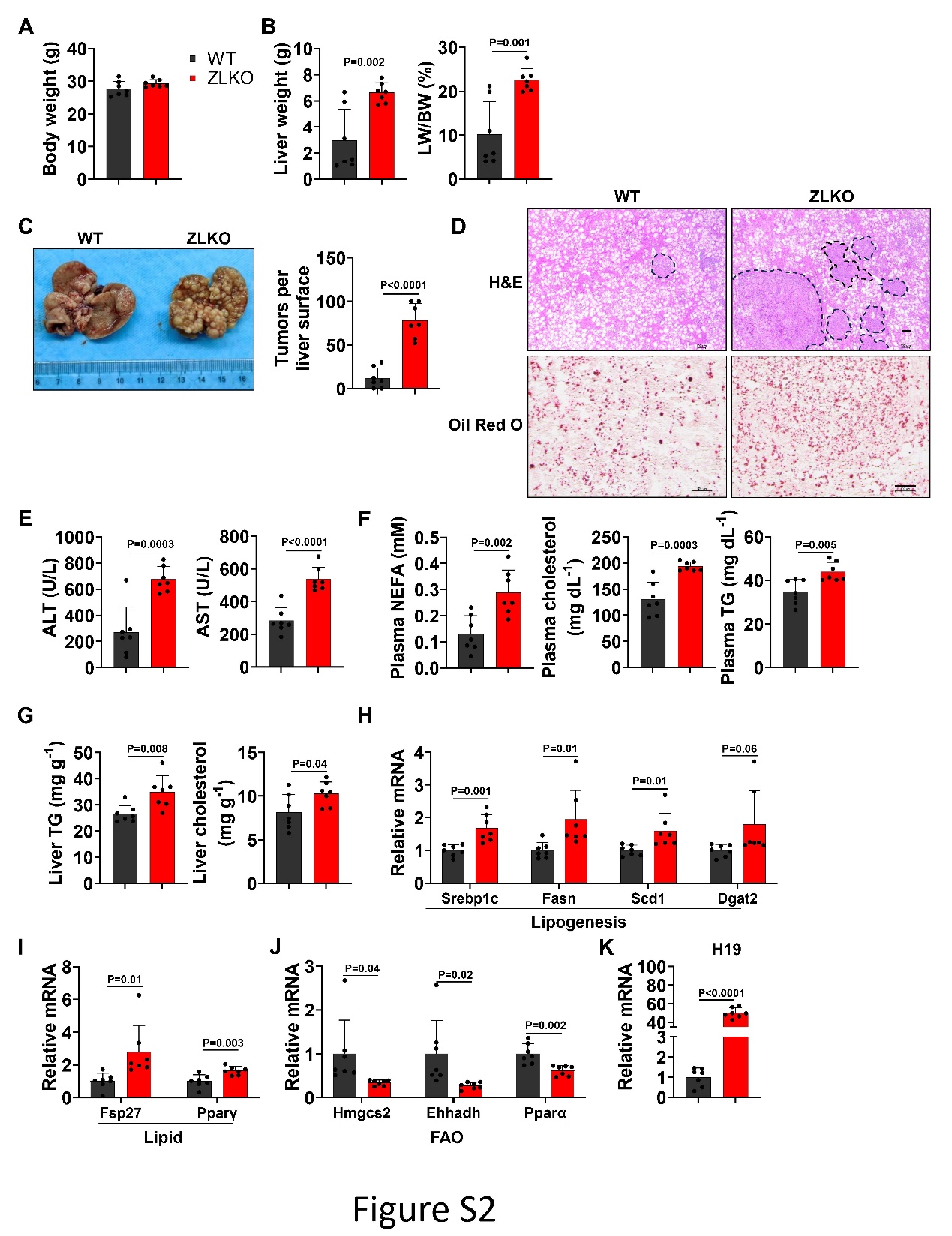


**Figure S2. Zbtb7b knockout promotes Akt/N-Ras-induced HCC.**

1. Body weights of AAV-TBG-control (WT, black, n=7) and AAV-TBG-Cre-injected Zbtb7b^flox/flox^ (ZLKO, red, n=7) mice after hydrodynamic transfection (HDT) of Akt/N-Ras vectors along with SB100 injected and fed with chow diet for four weeks.
2. Liver weights (left) and liver-to-body weight ratios (right) of WT and ZLKO mice after HDT of Akt/N-Ras vectors.
3. Representative liver images (left) of WT and ZLKO mice after HDT of Akt/N-Ras vectors. Column plot (right) shows the number of tumors per liver surface in WT and ZLKO mice.
4. H&E staining and Oil Red O staining of WT and ZLKO mice after HDT of Akt/N-Ras vectors (scale bar=100 μm).
5. Plasma ALT (left) and AST (right) levels in WT and ZLKO mice.
6. Plasma non-esterified fatty acid (NEFA, left), cholesterol (middle) and triglyceride (TG, right) levels in WT and ZLKO mice.
7. Liver TG (left) and cholesterol (right) contents in WT and ZLKO mice.

(H-J) QPCR analysis of hepatic genes involved in lipogenesis (H), lipid information (Lipid, I) and fatty acid oxidation (FAO, J) in AAV-TBG-control (WT) and AAV-TBG-Cre-injected Zbtb7b^flox/flox^ (ZLKO) mice after HDT of Akt/N-Ras vectors.

(K) QPCR analysis of H19 expression in WT and ZLKO mice after HDT of Akt/N-Ras vectors.

The data in A-C, E-K represent as mean ± SD. WT vs. ZLKO, two-tailed unpaired Student’s t-tests.


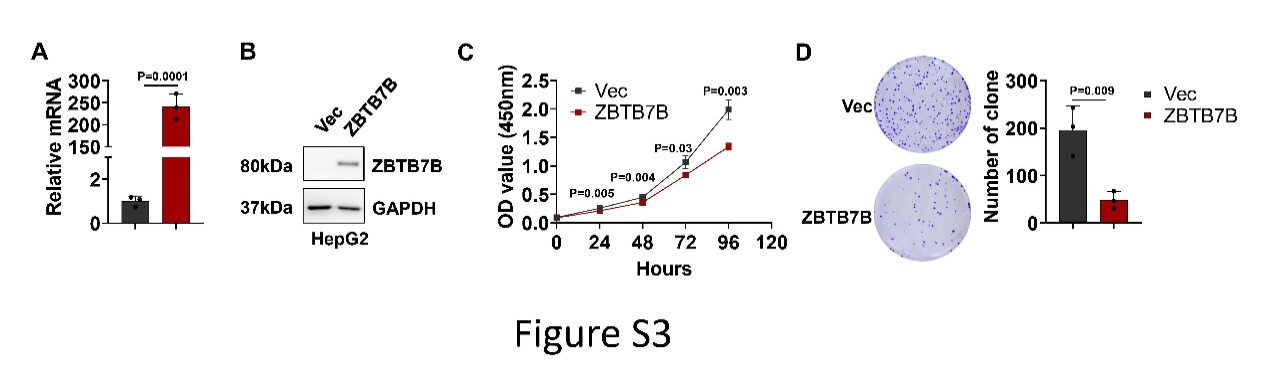


**Figure S3. Zbtb7b inhibits the proliferation of HepG2 cells.**

(A) QPCR analysis of the expression of ZBTB7B in Vec- (black, n=3) and ZBTB7B-overexpressing HepG2 cells (claret, n=3).

(B) Immunoblotting of lysates from vector (Vec) and ZBTB7B-overexpressing (ZBTB7B) HepG2 cells.

(C) The proliferation ability of Vec- and ZBTB7B-overexpressing HepG2 cells was measured via Cell Counting Kit‑8 (CCK-8).

(D) The colony formation ability of Vec- and ZBTB7B-overexpressing HepG2 cells was measured via colony formation assays.

The data in A, D are presented as the mean ± SD. Vec vs. ZBTB7B, two-tailed unpaired Student’s t-test. The data in C represent the mean ± SD. Vec vs. ZBTB7B, two-way ANOVA with multiple comparisons.


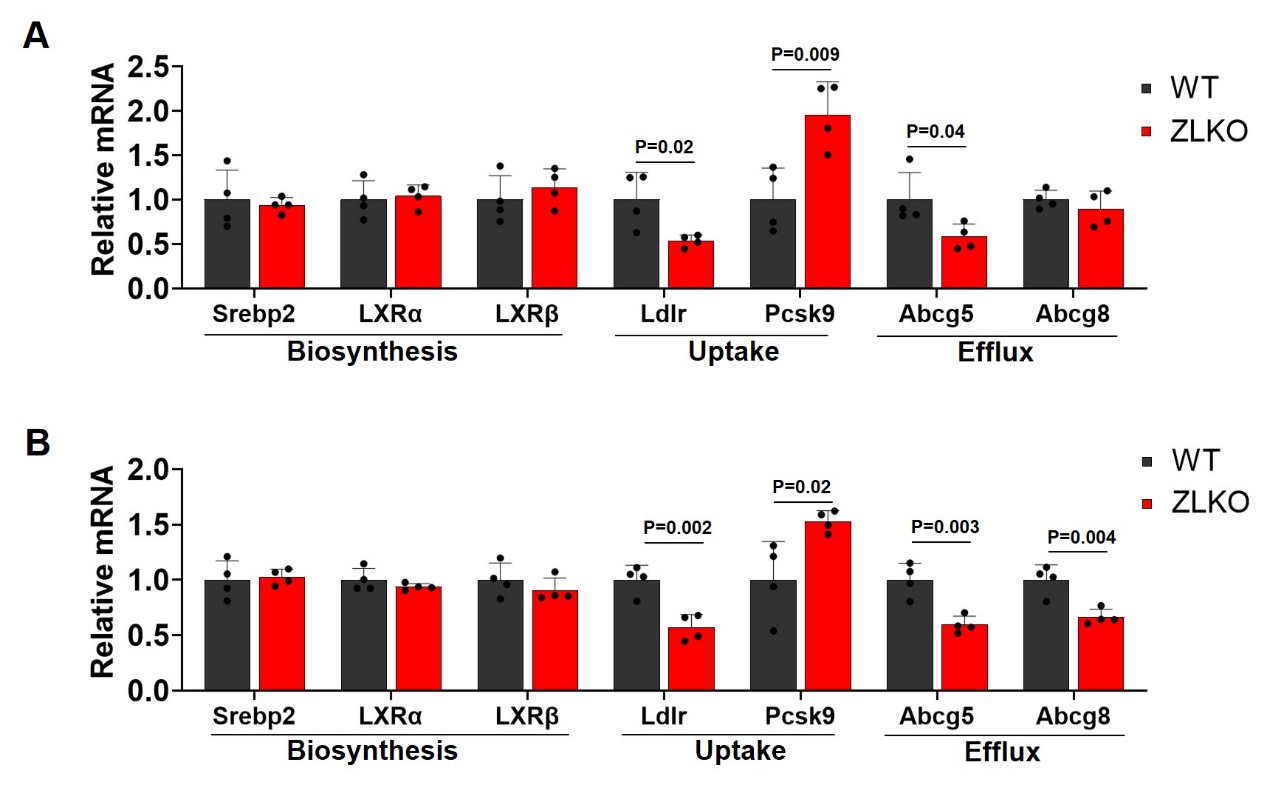


**Figure S4. Deficiency of Zbtb7b mediates cholesterol biosynthesis, uptake and efflux-related gene expression in hepatocytes.**

(A-B) QPCR analysis of hepatic genes involved in cholesterol biosynthesis, uptake and efflux in AAV-TBG-control (WT, black, n=4) and AAV-TBG-Cre-injected Zbtb7bflox/flox (ZLKO, red, n=4) mice after chow diet feeding for one week (A) and HFD feeding for 5 months (B). The data in A-B are presented as the mean ± SD, WT vs. ZLKO, two-tailed unpaired Student’s t test.

**Table S1. Primer lists of qPCR.**

| Mouse | Forward | Reverse |
| --- | --- | --- |
| Zbtb7b | CTCACCCATCCCTTGACCTA | CCAGCTCCTCTGGTGATAGC |
| H19 | GAACAGAAGCATTCTAGGCTGG | TTCTAAGTGAATTACGGTGGGTG |
| Srebp1c | GATGTGCGAACTGGACACAG | CATAGGGGGCGTCAAACAG |
| Fasn | GGAGGTGGTGATAGCCGGTAT | TGGGTAATCCATAGAGCCCAG |
| Scd1 | GCTGGAGTACGTCTGGAGGAA | TCCCGAAGAGGCAGGTGTAG |
| Dgat2 | GCGCTACTTCCGAGACTACTT | GGGCCTTATGCCAGGAAACT |
| PPARα | GCAGTGCCCTGAACATCGA | CGCCGAAAGAAGCCCTTAC |
| PPARγ | CTGACCCAATGGTTGCTGAT | GGTGGAGATGCAGGTTCTAC |
| Ehhadh | CAGATGAAGCACTCAAGCTTG | ACCTTGGCAATGGCTTCTGCA |
| Hmgcs2 | GACATCAACTCCCTGTGCCTG | GATGTCAGTGTTGCCTGAATC |
| Fsp27 | TCGACCTGTACAAGCTGAACCCT | AGGTGCCAAGCAGCATGTGACC |
| Col1a1 | AAGAGGCGAGAGAGGTTTCC | AGAACCATCAGCACCTTTGG |
| Col1a2 | GTAACTTCGTGCCTAGCAACA | CCTTTGTCAGAATACTGAGCAGC |
| Col3a1 | CTGTAACATGGAAACTGGGGAAA | CCATAGCTGAACTGAAAACCACC |
| Col5a2 | ACAGGTGAAGTGGGATTCTCA | CCATAGCACCCATTGGACCA |
| Col5a3 | CGGGGTACTCCTGGTCCTAC | GCATCCCTACTTCCCCCTTG |
| Col6a1 | CTGCTGCTACAAGCCTGCT | CCCCATAAGGTTTCAGCCTCA |
| Col6a2 | AAGGCCCCATTGGATTCCC | CTCCCTTCCGACCATCCGAT |
| Col6a3 | GCTGCGGAATCACTTTGTGC | CACCTTGACACCTTTCTGGGT |
| Ccl2 | AGGTCCCTGTCATGCTTCTG | TCTGGACCCATTCCTTCTTG |
| Ccl5 | TGCCCACGTCAAGGAGTATTT | TTCTCTGGGTTGGCACACACT |
| Il1β | GAAATGCCACCTTTTGACAGTG | TGGATGCTCTCATCAGGACAG |
| Il6 | AGTTGCCTTCTTGGGACTGA | TCCACGATTTCCCAGAGAAC |
| Srebp2 | TCAGCACCGCTCCGCAGACGAG | TACCGTCTGCACCTGCTGCTGG |
| LXRα | AGGCTCAAGCCACTTCGGTGTC | AGAAGGAGCGCCTGTTACACTG |
| LXRβ | ACGTCACCCACTATTAAGGAAG | GCTCATCCTCTGGCTCTAAGAT |
| Ldlr | GATGTCGACTGTGTTGACGGCTC | CTGACTTGTCCTTGCAGTCTGC |
| Pcsk9 | GAGACCCAGAGGCTACAGATT | AATGTACTCCACATGGGGCAA |
| Abcg5 | CCAGATTATGTGCATCTTAGGCA | CTGCTCAGAAAAACGTCGCT |
| Abcg8 | CTGTGGAATGGGACTGTACTTC | GTTGGACTGACCACTGTAGGT |

| Human | Forward | Reverse |
| --- | --- | --- |
| ZBTB7B | CTCACCCATCCCTTGACCTA | AGCTTAGGTAGGCCATCAGGT |
| H19 | CCCACAACATGAAAGAAATGGTGC | GCTTCACCTTCCAGAGCCGAT |
| SREBP1 | CGGAACCATCTTGGCAACAGT | CGCTTCTCAATGGCGTTGT |
| FASN | ACAGCGGGGAATGGGTACT | GACTGGTACAACGAGCGGAT |
| SCD | GCCCCTCTACTTGGAAGACGA | AAGTGATCCCATACAGGGCTC |
| HMGCS2 | AAGTCCCTTAGCCTTCTCTACC | CCTTCCTCACGCTTCGATCC |

**Table S2. Information of HCC patients**

| Number | Gender | Pathological diagnosis | Cirrhosis |
| --- | --- | --- | --- |
| 1 | Female | HCC | G2S3 |
| 2 | Female | HCC | G2S3 |
| 3 | Female | HCC | G1S4 |
| 4 | Male | HCC | G1S2 |
| 5 | Male | HCC | G1S3 |
| 6 | Male | HCC | G2S2 |
| 7 | Male | HCC | G2S3 |
| 8 | Male | HCC | G1S3 |
| 9 | Male | HCC | G0 |
| 10 | Male | HCC | G2S4 |
| 11 | Male | HCC | G2S4 |
| 12 | Male | HCC | G2S4 |
